# The future of AI regulation in drug development: a comparative analysis

**DOI:** 10.1093/jlb/lsaf028

**Published:** 2025-11-07

**Authors:** Gabriela Lenarczyk, Timo Minssen, Nicholson Price, Arti Rai

**Affiliations:** Center for Advanced Studies in Bioscience Innovation Law, Faculty of Law, University of Copenhagen, Karen Blixens Plads 16, 2300 Copenhagen, Denmark; Center for Advanced Studies in Bioscience Innovation Law, Faculty of Law, University of Copenhagen, Karen Blixens Plads 16, 2300 Copenhagen, Denmark; Center for Advanced Studies in Bioscience Innovation Law, Faculty of Law, University of Copenhagen, Karen Blixens Plads 16, 2300 Copenhagen, Denmark; University of Michigan Law School, 625 S State St, Ann Arbor, MI 48109, USA; Duke University School of Law, 210 Science Dr, Durham, NC 27708, USA

**Keywords:** artificial intelligence, AI governance, drug development, EMA, FDA, pharmaceutical innovation

## Abstract

As artificial intelligence (AI) transforms drug development, regulatory frameworks are evolving to oversee its implementation, particularly at the US Food and Drug Administration (FDA) and the European Medicines Agency (EMA). This paper makes three contributions to understanding emerging regulatory approaches. First, we offer a comparative analysis of how these agencies have responded to AI-driven advances, incorporating new US executive orders and the European Union (EU)’s AI Act. Second, we propose a novel analytical framework to understand regulatory divergence: the FDA’s flexible, dialog-driven model contrasts with the EMA’s structured, risk-tiered approach, reflecting broader institutional and political-economic differences. While the former encourages innovation via individualized assessment, it can create uncertainty about general expectations; by contrast, the EMA’s clearer requirements may slow early-stage AI adoption but provide more predictable paths to market. Third, we examine whether AI applications—spanning target identification, generative chemistry, and clinical trial ‘digital twins’—are mature enough for standardized regulation, particularly amid shifting US policies and the EU’s structured oversight regime. Our analysis reveals patterns of convergence on risk-based principles but persistent transatlantic implementation differences, compounded by diminished US engagement in international cooperation. We conclude that heightened regulatory uncertainty in the USA under a new administration’s ‘America First’ stance and more stable, formalized rules in Europe both pose opportunities and challenges to AI-driven innovation in drug development.

## I. INTRODUCTION

Artificial intelligence (AI) stands poised to revolutionize drug development, promising to dramatically compress the traditional decade-long path from molecular discovery to market approval. This technological revolution manifests across the entire drug development continuum, from AI systems identifying novel drug targets and predicting molecular properties to algorithms optimizing clinical trial design and monitoring patient safety.^1^ Early successes in drug candidate identification and molecular interaction prediction have already demonstrated AI’s potential to accelerate the development process while potentially reducing costs.^2^

The increasing sophistication of AI systems in drug development, however, presents novel challenges for regulatory oversight. While recent advances in deep learning have dramatically expanded AI’s capabilities, they have also introduced unprecedented complexity and opacity into the drug development process. These systems often function as ‘black boxes’, where the path from input to output resists straightforward interpretation or explanation.^3^ This is a particularly concerning characteristic in pharmaceutical development, since decisions based on such black box outputs can directly impact patient safety and public health. AI systems may inadvertently amplify errors or preexisting biases in their training data, raising questions about the generalizability of their insights across diverse patient populations.^4^ Moreover, the technical complexity of these systems, often protected as proprietary information, creates additional challenges for transparent validation and oversight.^5^ The deployment of AI in drug development raises fundamental questions about data quality, standardization, security,^6^ and the appropriate balance between automated analysis and expert judgment.^7^ The urgency of addressing these challenges is highlighted by emerging evidence suggesting that regulatory uncertainty is shaping—and potentially constraining—patterns of AI adoption across drug development stage. The technical oversight of AI in drug development requires well-calibrated frameworks that account for both the stage of development and the potential impact on patient safety.

These regulatory imperatives emerge within one of the most heavily regulated sectors of the global economy. The pharmaceutical industry has long operated within a complex web of national and international oversight, built upon decades of careful harmonization efforts. Since the establishment of the International Council for Harmonization of Technical Requirements for Pharmaceuticals for Human Use (ICH) in 1990, regulators have worked to standardize pharmaceutical development requirements across jurisdictions. However, this framework of international cooperation now faces unprecedented challenges, both from AI applications that defy traditional regulatory paradigms and from a US administration that has signaled less interest in sustained international collaboration.^8^

This article addresses the legal question of how regulatory agencies should adapt established oversight frameworks to AI-driven drug development. Specifically, it examines the US Food and Drug Administration’s (FDA’s) flexible, case-specific model and the European Medicines Agency’s (EMA’s) structured, risk-tiered approach and situates them within broader political–economic contexts. The paper makes three contributions: first, a comparative analysis of FDA and EMA oversight of AI; second, an analytical framework to explain regulatory divergence; and third, an evaluation of future trajectories, including scenarios of convergence, divergence, or regulatory friction. These contributions aim to clarify the legal challenges and opportunities posed by AI in drug development and to inform ongoing debates on international harmonization.

## II. UNDERSTANDING DIFFERENT AI ADOPTION PATTERNS

Evidence from recent global drug development data indicates that regulatory environments may be shaping AI adoption across stages of the pipeline. AI tools are widely used in early-stage discovery, where oversight is limited, but uptake in later phases remains more cautious.^9^ For example, 76 per cent of AI use cases involve molecule discovery, compared with only 3 per cent in areas such as clinical outcomes analysis.^10^ This imbalance appears to reflect not only technical considerations but also uncertainty about regulatory expectations, particularly in clinical settings. Although companies have the capability to deploy advanced AI—from automated high-throughput screening to AI-driven pharmacovigilance^11^—unclear validation frameworks may be discouraging their use in later stages.^12^

Although companies have the technical capability to deploy advanced AI—from automated high-throughput screening to AI-driven pharmacovigilance—unclear validation frameworks, especially in clinical settings, may be discouraging their use in later stages.

The challenge is especially visible in the rise of ‘digital twins’ in clinical trials. These computational replicas of patients or trial cohorts, built from clinical and real-world data, are designed to emulate control-arm outcomes for study design, monitoring, or analysis. By enabling virtual control groups, digital twins could lower trial costs and accelerate patient access to new therapies.^13^ Yet their use raises fundamental questions: how should regulators validate such models, and by what standards should their reliability be judged? Evaluation must balance traditional trial requirements with new considerations unique to AI-driven simulations.^14^ Digital twins therefore illustrate the broader problem: adapting regulatory frameworks to innovations that transform established drug development practices. Effective oversight will need to provide both clarity and flexibility without stifling innovation.^15^

This challenge is exacerbated by the jurisdictional nature of oversight in an inherently global industry. By fall 2024, the FDA has received over 500 submissions incorporating AI components across various stages of drug development,^16^ yet stakeholders report insufficient guidance about regulatory requirements for AI/ML (machine learning) applications, particularly in clinical phases.^17^ Similarly, while the EMA advocates for AI integration throughout the drug lifecycle, it acknowledges the need for clearer frameworks to assess AI’s impact on benefit–risk calculations.^18^

## III. CURRENT REGULATORY LANDSCAPES

Among Western regulatory frameworks for AI in drug development, the FDA and EMA’s approaches have been particularly influential and well documented. While both agencies acknowledge AI’s transformative potential in drug development, they have adopted different approaches to oversight, reflecting broader differences in their regulatory philosophies and institutional contexts.

### III.A. The EMA

The EMA’s approach to AI regulation in drug development, as articulated in its 2024 Reflection Paper,^19^ establishes a regulatory architecture that systematically addresses AI implementation across the entire drug development continuum. This framework reflects the European Union (EU)'s broader strategy of implementing comprehensive technological oversight while maintaining sector-specific requirements for pharmaceutical development.^20^

At its core, the EMA framework introduces a risk-based approach that focuses on ‘high patient risk’ applications affecting safety and ‘high regulatory impact’ cases where substantial influence on regulatory decision-making exists.^21^ This calibrated oversight system places explicit responsibility on clinical trial sponsors, marketing authorization applicants/holders, and manufacturers to ensure AI systems are fit for purpose and aligned with legal, ethical, technical, and scientific standards. The framework mandates adherence to EU legislation, Good Practice standards, and current EMA guidelines, creating a clear accountability structure.

In early development phases, the framework applies lower regulatory scrutiny to drug discovery applications with minimal direct patient impact while emphasizing data quality, representativeness, and the mitigation of bias and discrimination risks. However, for clinical development, particularly in pivotal trials, the requirements become more stringent. These include pre-specified data curation pipelines, frozen and documented models, and prospective performance testing. Notably, the framework prohibits incremental learning during trials, aiming to ensure the integrity of clinical evidence generation.^22^

The post-authorization phase allows for more flexible AI deployment while maintaining rigorous oversight. Here, the framework permits continuous model enhancement but requires ongoing validation and performance monitoring, integrated within established pharmacovigilance systems.^23^

Technical requirements under the framework are comprehensive, mandating three key elements: traceable documentation of data acquisition and transformation, explicit assessment of data representativeness, and strategies to address class imbalances and potential discrimination. The EMA expresses a clear preference for interpretable models but acknowledges the utility of black-box models when justified by superior performance. In such cases, the framework requires explainability metrics and thorough documentation of model architecture and performance.^24^

For regulatory engagement, the EMA establishes clear pathways through its Innovation Task Force for experimental technology, Scientific Advice Working Party consultations, and qualification procedures for novel methodologies. These mechanisms facilitate early dialogue between developers and regulators, particularly crucial for high-impact applications where regulatory guidance can significantly influence development strategy.^25^

The framework promotes the integration of data science competencies with traditional pharmaceutical expertise, thereby bolstering cross-functional oversight of AI applications. It encourages early regulatory interaction for high-impact applications and requires comprehensive documentation throughout the development and deployment lifecycle. Risk management becomes paramount, with requirements for systematic assessment, mitigation strategies, and ongoing performance monitoring.

While aligned with the EU’s broader AI Act^26^ and ethical guidelines for trustworthy AI,^27^ the framework maintains pharmaceutical sector specificity. This means that the EMA’s requirements complement, rather than duplicate, the EU’s overarching rules for AI. Notably, the Commission’s newly published Guidelines on prohibited AI practices—developed to clarify the AI Act’s risk-based classifications and provide insight into practices deemed unacceptable due to their risks to European values and fundamental rights—reinforce this layered approach.^28^ The EMA’s framework provides high-level guidance for AI integration across the drug lifecycle, acknowledging that many technical aspects await further regulatory elaboration. Taken together, these instruments can be understood as the EMA’s tacit endorsement of AI adoption in European drug development programs, albeit within a structured regulatory environment.

This regulatory architecture reflects the EU’s broader political–economic context, particularly its emphasis on harmonized market rules and precautionary regulation across member states. While the Reflection Paper sets out intended objectives of clarity and predictability, the framework’s additional documentation and validation requirements may extend innovation cycles and create compliance burdens for smaller actors. This mirrors broader patterns in EU technology regulation, where comprehensive oversight frameworks—exemplified by the General Data Protection Regulation (GDPR),^29^ and the AI Act^30^—as well as sector specific regulations such as the Medical Device Regulation (MDR)^31^ and the In Vitro Diagnostic Medical Device Regulation (IVDR),^32^ have sometimes created friction between regulatory rigor and competitive agility.^33^ The delayed adoption and implementation of the AI Act due to rapid developments in generative AI, which required ‘last-minute’ changes to the draft legislation exemplifies the drawbacks of such broadly applicable legislation. Moreover, particularly for small- and medium-sized enterprises (SMEs) using AI in drug development, high compliance thresholds and an increasingly complex regulatory eco-system can be difficult to navigate.^34^ Critics point to experiences in biotechnology, where stringent EU regulations contributed to research migration toward more permissive jurisdictions like the USA and China.^35^ The EU’s structured approach may prove advantageous as healthcare AI applications face mounting demands for demonstrable validation and trustworthiness. This potential advantage, however, remains contingent upon the regulatory framework’s capacity to adapt to rapidly evolving technological paradigms—a characteristic tension in EU technology governance that emerges throughout this discussion.

### III.B. The FDA

The FDA’s approach demonstrates a markedly different regulatory philosophy from its international counterparts, characterized by flexible, context-specific oversight that evolves through direct engagement with specific industry applications. While the agency provides informal guidance and emphasizes transparency, it has historically favored case-by-case evaluation over prescriptive frameworks. This preference is reflected across multiple domains, from medical device regulation to biosimilar interchangeability determinations, where the FDA has explicitly embraced individualized assessment as a means to adapt oversight to emerging technologies.^36^ Rather than relying on formal notice-and-comment rulemaking, the agency develops its regulatory approach primarily through non-binding guidance documents and iterative engagement with specific applications. This heavy reliance on informal guidance allows the FDA to maintain flexibility and avoid legal challenges while still providing direction to industry, though critics argue this approach reduces accountability and regulatory certainty.^37^ The resulting regulatory framework remains inherently case-specific, with the FDA retaining broad discretion to deviate from its published guidance based on individual circumstances.^38^

This approach is embodied in the FDA’s Center for Drug Evaluation and Research (CDER) through multiple coordinated initiatives. The CDER AI Steering Committee serves as the central coordinating body, working in concert with complementary programs such as the Innovative Science and Technology Approaches for New Drugs Pilot Program and the Model-Informed Drug Development Pilot Program.

In June 2025, the FDA deployed an agency-wide large language model assistant (‘Elsa’) to support internal scientific review and inspection planning.^39^ According to the agency, Elsa can summarize adverse events, perform rapid label comparisons, assist with clinical-protocol review, and generate code for internal databases. It operates within a secure GovCloud environment without training on sponsor submissions. Elsa appears to be primarily an operational tool rather than a substantive regulatory change, and its use may therefore incrementally shorten review timelines and further incentivize well-structured, machine-readable submission materials that facilitate AI-assisted review. Elsa’s deployment does not purport to alter the agency’s risk-based approach to evaluating AI used in drug development.

This regulatory framework operates within a broader ecosystem of federal initiatives. The National Institute of Standards and Technology (NIST)’s AI Safety Institute has emerged as a crucial partner, developing technical guidance for biotechnology research and development (R&D) and regulatory rulemaking, including standards for computer-generated compounds. The AI Safety Institute builds upon NIST’s earlier work establishing federal engagement in AI technical standards development, creating a layered approach to oversight that maintains flexibility while ensuring rigorous standards.^40^ However, with the US transition to a new administration in 2025, the continuity and evolution of these initiatives remain to be determined, introducing an element of uncertainty into the longer-term regulatory landscape for AI in drug development.

The FDA’s 2023 discussion paper crystallized this regulatory philosophy, introducing a risk-based framework that emphasizes three interconnected domains: human-led governance with clear accountability and transparency; quality and reliability of data; and robust model development, performance, and validation protocols.^41^ Rather than establishing comprehensive requirements upfront, the FDA has opted for an iterative approach, developing guidance through direct engagement with industry applications. Industry response to this framework, evidenced through extensive public commentary, reveals broad support for the FDA’s risk-calibrated approach.^42^ Stakeholders particularly appreciate the emphasis on context-specific oversight rather than universal requirements. However, this feedback also illuminates areas requiring additional regulatory clarity, particularly regarding technical guidance for specific applications such as clinical trials and pharmacovigilance, and protocols for managing continuously learning AI systems. Recent developments suggest continued evolution of this regulatory framework. During an August 2024 workshop, Dr Cavazzoni, the Director of CDER, announced the FDA’s intention to develop more detailed risk-based guidance, indicating a measured progression toward more specific oversight while maintaining the core philosophy of flexible, context-dependent regulation.^43^

Recent US political developments complicate the FDA’s evolving stance. The transition to the Trump administration in January 2025 brought Executive Order 14148,^44^ which rescinds President Biden’s EO 14110.^45^ This new order mandates a review—and possible revision—of all AI-related policies, including the FDA’s January 2025 draft guidance on AI-driven drug development.^46^ Under Executive Order 14148, agencies must reevaluate regulatory actions that might ‘impede US AI leadership’. Consequently, this policy emphasis could weaken or delay the FDA’s 2025 guidance, which notably reflects the Agency’s aim, under prior leadership, to standardize a risk-based, context-of-use framework for validating AI models that generate data for regulatory decisions​. Thus, although we continue to consider this guidance as reflecting the FDA’s recent thinking, we do note the substantial potential for change going forward.

The 2025 guidance builds on previous discussion papers and underscores the FDA’s intent to move from purely ad hoc oversight toward a more formal methodology for evaluating AI reliability, while still retaining flexibility for emerging technologies: the Agency emphasizes ensuring ‘model credibility’—sponsors must demonstrate that an AI tool is reliable for its intended use, similar to how FDA reviewers already assess AI components in submissions​.^47^

At the same time, the FDA’s approach reflects the distinctive characteristics of the US regulatory landscape, where institutional frameworks emphasize market-driven innovation and administrative flexibility. Operating within a system of active Congressional oversight, the FDA has developed what might be characterized as ‘artisanal regulation’—a highly customized approach to oversight that responds precisely to specific contexts and applications. This individualized regulatory engagement emerges partly from an environment of intense public scrutiny of regulatory actions, where each decision must be carefully calibrated to balance innovation with public health protection. Although this methodology allows for nuanced evaluation of emerging technologies and promotes innovation through targeted guidance rather than broad prescriptive frameworks, it raises concerns that the Agency’s evolving stance could stray too far from its recent trajectory, which sought greater structure and predictability. If political pressures favor a more protectionist outlook, the resulting shifts may complicate ex ante clarity, industry-wide standardization, and international harmonization efforts, placing the FDA’s delicate balance between flexibility and formal oversight at renewed risk.

## IV. DIVERGING APPROACHES TO OVERSIGHT

The FDA and EMA’s approaches to AI regulation in drug development reflect fundamentally different regulatory philosophies while pursuing the same core objective of ensuring safe and effective AI implementation in drug development.

The FDA’s approach demonstrates a flexible, context-specific oversight methodology that evolves through direct engagement with industry applications. The Agency employs what their discussion paper terms a ‘multifaceted approach to enhance mutual learning and establish dialogue with stakeholders’.^48^ This approach facilitates targeted guidance rather than broad prescriptive frameworks and is operationalized through the abovementioned, multiple coordinated initiatives within CDER. The EMA, while maintaining its own active channels for industry dialogue and learning, has established a more structured regulatory architecture in its 2024 Reflection Paper, setting explicit requirements across different development phases.

Both agencies emphasize risk-based approaches but implement them differently. The FDA’s discussion paper advocates for ‘measures commensurate with the level of risk posed by the specific context of use’^49^ without prescribing specific risk levels. The EMA explicitly defines risk categories and corresponding requirements, stating that ‘the level of scrutiny depends on the level of risk and regulatory impact posed by the system’.^50^

These differences reflect deeper divergences in regulatory philosophies and political economy considerations. Relative to the EMA, FDA’s approach embodies a greater emphasis on market-driven innovation and administrative flexibility, treating regulation as an evolving dialogue between industry and regulators—a stance shaped by intense Congressional and judicial oversight and increasing public scrutiny of regulatory processes, which may incentivize less formal, individualized engagement with sponsors. Although the FDA has used informal, context-specific approaches for decades, the agency may be even more inclined to use these approaches going forward. In particular, the US Supreme Court has, over the last few years, substantially increased its scrutiny of agency rules adopted through more formal processes.^51^ Accordingly, fleshing out policies through individual decisions may help to shield the agency from judicial challenges.

In contrast, the EMA’s framework mirrors the EU’s broader regulatory culture, prioritizing predictability and harmonized standards across member states as a means to build market confidence, reflecting its mandate to balance member state interests within the EU’s comprehensive regulatory framework. The practical implications of these approaches are evident in their implementation: while the EMA requires extensive upfront documentation and validation, potentially creating higher initial barriers but clearer pathways, the FDA’s case-by-case approach offers lower initial barriers but demands ongoing dialogue and adaptation, potentially creating uncertainty for novel applications.

These contrasting approaches also carry implications for innovation and market dynamics. The EU’s structured framework may favor established companies with resources to navigate comprehensive requirements, while potentially limiting smaller innovators’ market entry. This dynamic potentially threatens not just SMEs—which form the backbone of the EU economy^52^—but also the broader innovation ecosystem that larger companies depend on for their own growth and development. Conversely, the US strategy’s flexibility might better accommodate rapid innovation but creates challenges for companies seeking to scale AI applications globally. These differences create particular challenges for harmonization efforts, as companies must reconcile fundamentally different regulatory philosophies and requirements.^53^ It may also be harder simply to know exactly what the contours of the policy are under the USA’s approach, given the inherent specificity and nontransparency of private, individual agency-firm discussions.

Though formal bilateral harmonization efforts between the agencies are still emerging. Both agencies participate in broader international initiatives, such as the International Coalition of Medicines Regulatory Authorities (ICMRA), which, under EMA’s leadership, has released recommendations for navigating AI opportunities and challenges in drug development.^54^

## V. DISCUSSION

Current evidence reveals encouraging patterns of regulatory convergence in AI oversight for drug development. The FDA and EMA’s emerging frameworks demonstrate fundamental alignment in core principles - particularly in risk-based assessment and innovation-conscious oversight – while differing primarily in implementation methodologies. These differences reflect broader regulatory philosophies: the FDA’s flexible, case-specific oversight contrasts with the EMA’s structured and comprehensive approach.

As regulatory frameworks evolve, questions arise about how to balance flexibility and standardization in overseeing rapidly advancing AI technologies. This tension is not unique to drug development but reflects broader patterns in AI governance. Gasser and Mayer-Schönberger’s concept of ‘guardrails’ provides a useful lens here, emphasizing the need for frameworks that maintain human oversight while leveraging AI capabilities.^55^ Their analysis suggests that neither complete reliance on AI autonomy nor overly rigid human control is effective; instead, successful governance lies in adaptive structures that integrate core principles with flexibility to address jurisdictional needs.

In the context of drug development, effective implementation of the guardrails concept requires, at a minimum, interoperability between oversight methodologies of the FDA and EMA. For instance, the FDA’s iterative, case-specific approach could complement the EMA’s structured requirements, fostering cross-border learning and collaboration.^56^ By building such bridges, regulatory diversity can become a source of resilience and innovation, enabling jurisdictions to retain distinct oversight approaches while working toward shared goals. Through this approach, interoperable frameworks could accelerate AI-driven drug development by reducing friction and ensuring that safety, efficacy, and trust remain central.

However, implementing such interoperability presents challenges that will likely shape the future of AI regulation in drug development. Three potential scenarios emerge. In a ‘pragmatic convergence’ scenario, companies might default to meeting the EMA’s more structured requirements, knowing that such compliance will likely satisfy the FDA’s case-by-case assessment. In this scenario, the EMA could emerge as the de facto standard-setter, similar to how the EU’s GDPR has influenced global data protection practices. While pragmatic convergence could streamline compliance for some companies, it may inadvertently favor larger players capable of navigating the EMA’s rigorous standards, potentially disadvantaging smaller innovators.

Alternatively, ‘strategic divergence’ could emerge, where companies pursue parallel development paths optimized for each jurisdiction. Strategic divergence might involve using AI tools more extensively in FDA submissions while maintaining traditional development approaches for EMA applications. Although strategic divergence would allow companies to leverage the strengths of each regulatory system, it introduces inefficiencies and higher costs, particularly for global market entry.

The third and most concerning scenario would involve increasing regulatory friction, where divergent approaches create cumulative barriers to AI adoption. This could particularly impact smaller companies lacking resources to navigate multiple regulatory frameworks, potentially stifling innovation where AI might offer the greatest efficiency gains.

These scenarios underscore the practical importance of establishing effective guardrails for AI in drug development. By aligning on foundational principles while allowing for jurisdictional adaptations, regulators can avoid unnecessary barriers and foster innovation. Interoperable frameworks, inspired by successful models like the ICH, can enable jurisdictions to maintain their autonomy while promoting global collaboration. Yet the potential changes in direction under the Trump administration—particularly its stated focus on ‘domestic innovation’—could complicate efforts to forge a unified approach across borders, further intensifying the need for robust but flexible guardrails. Achieving this balance will not only accelerate AI adoption but also ensure that new therapies reach patients worldwide without compromising safety, efficacy, or trust.

## Footnotes

1 US Food & Drug Admin., *Using Artificial Intelligence and Machine Learning in the Development of Drug and Biological Products: Discussion Paper and Request for Feedback* (2024), https://www.fda.gov/media/167973/download (accessed June 2, 2025), at II.

2 Janet Freilich & Arti Rai, *What Patents on AI-Developed Drugs Reveal*, 388 Science 924 (2025); Feng Ren et al*., AlphaFold Accelerates Artificial Intelligence-Powered Drug Discovery: Efficient Discovery of a Novel CDK20 Small-Molecule Inhibitor*, 14 Chem. Sci. 1443 (2023); Andreas Bender & Isidro Cortés-Ciriano, *Artificial Intelligence in Drug Discovery: What Is Realistic, What Are Illusions? Part 1: Ways to Make an Impact, and Why We Are Not There Yet*, 26 Drug Discovery Today 511 (2021).

3 Marco Tulio Ribeiro, Sameer Singh and Carlos Guestrin, *Why Should I Trust You?’ Explaining the Predictions of Any Classifier*. Proceedings of the 22nd ACM SIGKDD International Conference on Knowledge Discovery and Data Mining, San Francisco, 13–17 August 2016, 1135–1144.

4 Emilio Ferrara, *The Butterfly Effect in Artificial Intelligence Systems: Implications for AI Bias and Fairness*, 15 Machine Learning With Applications 100,525 (2024).

5 World Health Organization, *Benefits and Risks of Using Artificial Intelligence for Pharmaceutical Development and Delivery: Discussion Paper* (2024), https://www.who.int/publications/i/item/9789240088108 (accessed June 2, 2025).

6
*Id.*

7 Sarfaraz K. Niazi, *The Coming of Age of AI/ML in Drug Discovery, Development, Clinical Testing, and Manufacturing: The FDA Perspectives*, 17 Drug Des. Devel. Ther. 2691 (2023).

8 Haider J. Warraich, Troy Tazbaz & Robert M. Califf, *FDA Perspective on the Regulation of Artificial Intelligence in Health Care and Biomedicine*, Jama (15 Oct. 2024), https://jamanetwork.com/journals/jama/fullarticle/2825146 (accessed October 27, 2025).

9 US Government Accountability Office. *Artificial Intelligence in Health Care: Benefits and Challenges of Machine Learning in Drug Development*, GAO-20-215SP (2019), https://www.gao.gov/assets/gao-20-215sp.pdf (accessed October 27, 2025); Louise C. Druedahl et al*.*, *Use of Artificial Intelligence in Drug Development*, 7 Jama Network Open e241230 (2024).

10 Druedahl et al., *Id.*

11 Ashfaq U. Rehman et al., *Role of Artificial Intelligence in Revolutionizing Drug Discovery*, Fundamental Research (9 May 2024), https://www.sciencedirect.com/science/article/pii/S266732582400205X (accessed October 27, 2025).

12 Druedahl et al, *supra* note 9.

13 Gen Li, *A New Regulatory Road in Clinical Trials: Digital Twins*, Applied Clinical Trials (12 Sept. 2024), https://www.appliedclinicaltrialsonline.com/view/new-regulatory-road-clinical-trials-digital-twins (accessed October 27, 2025).

14 Eg EU Clinical Trials Regulation No 536/2014, especially Articles 28–29 (trial design & control groups); 21 C.F.R. § 312 (Investigational New Drug application requirements—trial design, control arms, data integrity; novel considerations specific to AI-driven simulations—*see Id.*

15 Kang Zhang, Xin Yang, Yifei Wang et al., *Artificial Intelligence in Drug Development*, 31 Nat. Med. 45 (2025).

16 Katie Palmer, *Q&A: How the FDA Is Approaching AI in Clinical Trials and Drug Development*, Stat Health Tech (9 Oct. 2024), https://www.statnews.com/2024/10/09/fda-regulation-ai-clinical-trials-drug-discovery-tala-fakhouri/ (accessed October 27, 2025).

17
*Comments to the U.S. Food & Drug Admin.’s Discussion Paper Using Artificial Intelligence and Machine Learning in the Development of Drug and Biological Products*, Docket No. FDA-2023-N-0743, https://www.regulations.gov/docket/FDA-2023-N-0743 (accessed June 2, 2025).

18 European Federation of Pharmaceutical Industries and Associations. *EFPIA Position on the Use of Artificial Intelligence in the Medicinal Product Lifecycle* (2024), https://www.efpia.eu/media/tzeavw1t/efpia-position-on-the-use-of-artificial-intelligence-in-the-medicinal-product-lifecycle.pdf (accessed October 27, 2025).

19 European Medicines Agency (EMA), *Reflection Paper on the Use of Artificial Intelligence (AI) in the Medicinal Product Lifecycle*, EMA/CHMP/CVMP/83833/2023 (2024).

20 The EU AI Act introduces a risk-based regulatory framework for AI systems, categorizing applications based on their risk level and establishing requirements to ensure safety, transparency, and accountability. *See* Regulation of the European Parliament and of the Council of 13 June 2024 Laying Down Harmonized Rules on Artificial Intelligence and Amending Certain Union Legislative Acts, OJ L, 2024/1689 (AI Act).

21 EMA, *supra* note 16, at 4.

22
*Id.*

23
*Id.* at 10.

24
*Id.* For illustration, when AI systems inform pivotal trials the protocol and SAP should pre-specify model inputs, training/validation datasets, model freeze, and prospectively defined performance/interpretability metrics, in line with ICH E6(R2) Good Clinical Practice (eg §§2.10, 5.5) and ICH E9 (eg §§2.1–2.2 on pre-specification and statistical principles), as well as the EU Clinical Trials Regulation (Regulation (EU) No 536/2014), Annex I on protocol content and dossier documentation*.*

25 In March 2025, the EMA’s human medicines committee (CHMP) issued its first Qualification Opinion for an AI tool (AIM-NASH) used in MASH clinical trials, thereby signaling acceptance of AI-based evidence within the Agency’s structured oversight framework.

26 AI Act, *supra* note 17.

27 Independent High-Level Expert Group on Artificial Intelligence, *Ethics Guidelines for Trustworthy Artificial Intelligence* (8 Apr. 2019), https://ec.europa.eu/newsroom/dae/document.cfm?doc_id=60419 (accessed October 27, 2025).

28 Note that the European AI Office is also currently developing the first General-Purpose AI Code of Practice (CoP) that will detail the AI Act rules for providers of AI models. The final version of the first CoP is to be published after May 2025. The European Commission, Annex to the Communication to the Commission. Approval of the content of the draft Communication from the Commission—Commission Guidelines on prohibited artificial intelligence practices established by Regulation (EU) 2024/1689 (AI Act), C(2025) 884 final.

29 Regulation (EU) 2016/679 of the European Parliament and of the Council of 27 Apr. 2016, 2016 O.J. (L 119) 1 (General Data Protection Regulation).

30 AI Act, *supra* note 17.

31 Regulation (EU) 2017/745 of the European Parliament and of the Council of 5 Apr. 2017 on Medical Devices, 2017 O.J. (L 117) 1 (amended 9 July 2024).

32 Regulation (EU) 2017/746 of the European Parliament and of the Council of 5 Apr. 2017 on In Vitro Diagnostic Medical Devices, 2017 O.J. (L 117) 176 (amended 9 July 2024).

33
*See broadly*  Anu Bradford, The Brussels Effect: How the European Union Rules the World (Oxford Univ. Press 2019).

34 Mateo Aboy, Timo Minssen & Effy Vayena, *Navigating the EU AI Act: Implications for Regulated Digital Medical Products*, 7 Npj Digit. Med. 237 (2024); Sebastian Porsdam Mann, Glenn Cohen & Timo Minssen, *The EU AI Act: Implications for U.S. Health Care*, 1(11) New Eng. J. Med. Ai (2024).

35 Communication from the Commission to the European Parliament, the Council, the European Economic and Social Committee and the Committee of the Regions, *Building the Future with Nature: Boosting Biotechnology and Biomanufacturing in the EU*, COM(2024) 137 final (20 Mar. 2024).

36 US Food & Drug Admin., *Deciding When to Submit a 510(k) for a Change to an Existing Device: Guidance for Industry and FDA Staff* (2017), https://www.fda.gov/media/98657/download (accessed October 27, 2025); US Food & Drug Admin., *Considerations in Demonstrating Interchangeability with a Reference Product: Guidance for Industry* (2019), https://www.fda.gov/media/124907/download (accessed October 27, 2025).

37 Kevin M. Lewis, *Informal Guidance and the FDA*, 66 Food & Drug L.J. 507 (2011).

38 In general, FDA guidance documents do not establish legally enforceable responsibilities and are not binding on the FDA or the public, allowing the agency broad discretion to deviate where justified. US Food & Drug Admin., *Guidances* (database), https://www.fda.gov/regulatory-information/search-fda-guidance-documents (accessed June 2, 2025).

39 US Food & Drug Admin., *FDA Launches Agency-Wide AI Tool to Optimize Performance for the American People* (2025). https://www.fda.gov/news-events/press-announcements/fda-launches-agency-wide-ai-tool-optimize-performance-american-people (accessed October 27, 2025).

40 Executive Order 13960 further reinforced this approach by establishing principles for federal agencies’ use and regulation of AI technologies and was complemented by Executive Order 14110, which expands the federal focus on AI governance by mandating enhanced safety, security, and equity measures across AI development and deployment. Exec. Order No. 13,960, Promoting the Use of Trustworthy Artificial Intelligence in the Federal Government, 85 Fed. Reg. 78,939 (3 Dec. 2020); Exec. Order No. 14,110, Safe, Secure, and Trustworthy Development and Use of Artificial Intelligence, 88 Fed. Reg. 75,191 (30 Oct. 2023).

41 US Food & Drug Admin., *supra* note 1.

42 Comments to the US Food & Drug Admin.’s Discussion Paper, *supra* note 14.

43 Benjamin M. Zegarelli, Joanne S. Hawana & Matthew Tikhonovsky, *FDA and CTTI Hold Joint Workshop on AI in Drug Development—AI: The Washington Report,*  Mintz Viewpoints (9 Aug. 2024), https://www.mintz.com/insights-center/viewpoints/2791/2024-08-09-fda-and-ctti-hold-joint-workshop-ai-drug-development-ai (accessed October 27, 2025).

44 Exec. Order No. 14,148, Initial Rescissions of Harmful Executive Orders and Actions, https://www.govinfo.gov/content/pkg/FR-2025-01-28/pdf/2025-01901.pdf (accessed October 27 2025).

45 Exec. Order No. 14,110, *supra* n. 40.

46 US Food & Drug Admin., *Considerations for the Use of Artificial Intelligence to Support Regulatory Decision-Making for Drug and Biological Products: Draft Guidance for Industry and Other Interested Parties* (2025), https://www.fda.gov/regulatory-information/search-fda-guidance-documents/considerations-use-artificial-intelligence-support-regulatory-decision-making-drug-and-biological (accessed October 27, 2025).

47
*Id.*

48 US Food & Drug Admin*, supra* note 1.

49
*Id.*

50 EMA, *supra* note 16, at 4.

51
*Loper Bright Enters. v. Raimondo*, 603 U.S. 369 (2024); *West Virginia v. EPA*, 603 U.S. 279 (2024).

52 SMEs represent 99% of all EU businesses. *See* Communication from the Commission to the European Parliament, the Council, the European Economic and Social Committee and the Committee of the Regions, SME Relief Package, COM(2023) 535 final (12 Sept. 2023).

53 Urs Gasser, *Governing AI with Intelligence*, 40(4) Issues in Sci. & Tech. 36 (2024).

54 International Coalition of Medicines Regulatory Authorities, *Horizon Scanning Assessment Report—Artificial Intelligence* (6 Aug. 2021), https://www.icmra.info/drupal/sites/default/files/2021-08/horizon_scanning_report_artificial_intelligence.pdf (accessed October 27, 2025).

55
Urs Gasser & Viktor Mayer-Schönberger, Guardrails: Guiding Human Decisions In The Age OF AI (Princeton Univ. Press 2024).

56 The July 2025 release of America’s AI Action Plan, which recommends regulatory sandboxes and Centers of Excellence supported by agencies such as the FDA, suggests a broader US policy interest in fostering AI-enabled innovation. While this initiative is noteworthy, its emphasis appears to fall primarily on AI-enabled medical devices and general innovation ecosystems, rather than directly altering FDA’s emerging approach to drug development. *See* The White House, *Winning the Race. America’s AI Action Plan*, (July 2025), https://www.whitehouse.gov/wp-content/uploads/2025/07/Americas-AI-Action-Plan.pdf (accessed October 27, 2025).

## Notes

**Dr Gabriela Lenarczyk** is a post-doctoral fellow at the Centre for Advanced Bioscience Innovation Law (CeBIL), University of Copenhagen, where she examines how pharmaceutical IP rights, regulatory exclusivities, and clinical-trial data protection shape sustainable access to medicines. A Polish-qualified attorney, she earned her PhD in Law (summa cum laude) from the Polish Academy of Sciences following earlier studies at Jagiellonian University and the London School of Economics. Her 2024 monograph on patent pledges and recent articles in *npj Digital Medicine*, *Journal of Intellectual Property Law & Practice*, and *IIC* probe open-innovation tools, AI-enabled trials, and EU data-transparency reform. Lenarczyk was a 2023–24 Thomas Edison Innovation Fellow with George Mason’s C-IP^2^ and now advises the Horizon-funded CREATIC gene-therapy consortium on IP strategy. Named a ‘Rising Star’ by Wolters Kluwer Poland in 2024.

**Professor Timo Minssen** directs the University of Copenhagen’s Center for Advanced Studies in Bioscience Innovation Law (CeBIL) and holds the UNESCO Co-Chair on the Right to Science. A German-Swedish jurist, he earned LL.M., Licentiate and doctoral degrees in IP law at Lund and Uppsala after training at Göttingen, the Max Planck Institute for Innovation & Competition, the European Patent office, law firms, and courts. His 230-plus publications integrate patent, competition, and regulatory analysis for technologies from CRISPR to large-language-model diagnostics, including *JAMA* and *Science* pieces on AI in medicine. Minssen advises the WHO, WIPO, and the European Commission and serves on multiple international ethics and policy committees. He also holds affiliate appointments at Harvard Law School’s Petrie-Flom Center, Cambridge University, and McGill’s Centre of Genomics & Policy and is a frequent visiting professor at Oxford, Munich, and Waseda.

**Professor W. Nicholson Price** II teaches at the University of Michigan Law School, where he explores how law channels innovation in the life sciences, especially big data and AI-driven medicine. He holds a BA from Harvard plus a PhD in Biological Sciences and JD from Columbia and clerked for Judge Carlos T. Bea on the US Court of Appeals for the Ninth Circuit. After fellowships at Harvard’s Petrie-Flom Center and UC Hastings, he taught at New Hampshire before joining Michigan in 2018. His work—featured in *Science, Nature,* and *NEJM*—tackles patent disclosure, data access, and algorithmic governance. He co-leads the Precision Medicine, AI & Law Project, is a senior fellow with Copenhagen’s CeBIL, and speaks worldwide on health-tech regulation.

**Professor Arti K. Rai** holds the Elvin R. Latty Chair at Duke Law and co-directs its Center for Innovation Policy. A leading voice on patent, administrative, and health-innovation law, from 2009 to 2010, she headed the Office of Policy and International Affairs at the US Patent and Trademark Office, helping craft the post-grant review system later enacted in the America Invents Act. Rai earned a BA in biochemistry/history and a JD cum laude from Harvard, then practiced at Jenner & Block and the US Department of Justice. Her scholarship on patent quality, biosimilar ‘thickets’, and AI-enabled drug discovery appears in *Nature Biotechnology, Health Affairs,* and top law reviews. She is a member of multiple distinguished councils and has served as a member of the National Advisory Council for Human Genome Research, as a public member of the Administrative Conference of the United States, and on numerous National Academies committees.

